# Hypoxia-Induced HIF-1α Expression Promotes Neurogenic Bladder Fibrosis via EMT and Pyroptosis

**DOI:** 10.3390/cells11233836

**Published:** 2022-11-29

**Authors:** Qi Li, Yifan Hong, Jing Chen, Xiazhu Zhou, Xiaomao Tian, Yihang Yu, Lianju Shen, Chunlan Long, Miao Cai, Shengde Wu, Guanghui Wei

**Affiliations:** 1 Department of Urology, Children’s Hospital of Chongqing Medical University, Chongqing 400014, China; 2 Chongqing Key Laboratory of Children Urogenital Development and Tissue Engineering, Ministry of Education Key Laboratory of Child Development and Disorders, National Clinical Research Center for Child Health and Disorders, China International Science and Technology Cooperation Base of Child Development and Critical Disorders, Chongqing Key Laboratory of Pediatrics, Chongqing 400014, China

**Keywords:** spinal cord injury, bladder function, fibrosis, HIF-1α, pyroptosis

## Abstract

Background: Neurogenic bladder (NB) patients exhibit varying degrees of bladder fibrosis, and the thickening and hardening of the bladder wall induced by fibrosis will further affect bladder function and cause renal failure. Our study aimed to investigate the mechanism of bladder fibrosis caused by a spinal cord injury (SCI). Methods: NB rat models were created by cutting the bilateral lumbar 6 (L6) and sacral 1 (S1) spinal nerves. RNA-seq, Western blotting, immunofluorescence, cell viability and ELISA were performed to assess the inflammation and fibrosis levels. Results: The rats showed bladder dysfunction, upper urinary tract damage and bladder fibrosis after SCI. RNA-seq results indicated that hypoxia, EMT and pyroptosis might be involved in bladder fibrosis induced by SCI. Subsequent Western blot, ELISA and cell viability assays and immunofluorescence of bladder tissue confirmed the RNA-seq findings. Hypoxic exposure increased the expression of HIF-1α and induced EMT and pyroptosis in bladder epithelial cells. Furthermore, HIF-1α knockdown rescued hypoxia-induced pyroptosis, EMT and fibrosis. Conclusion: EMT and pyroptosis were involved in the development of SCI-induced bladder fibrosis via the HIF-1α pathway. Inhibition of the HIF-1α pathway may serve as a potential target to alleviate bladder fibrosis caused by SCI.

## 1. Introduction

Neurogenic bladder (NB) is a dysfunction of the bladder or urethra due to a variety of neurological disorders. A common cause of NB in children is myelodysplasia, including spina bifida and meningocele [1]. NB presents with various bladder dysfunctions, including increased intravesical pressure, poor compliance, bladder wall thickening, vesicoureteral reflux and eventually renal failure [2]. With the progression of the disease, NB patients exhibit varying degrees of bladder fibrosis and the thickening and hardening of the bladder wall caused by fibrosis further impact bladder and renal function. The main treatment methods for NB include clean intermittent catheterization (CIC), oxybutynin and surgical intervention, which can relieve the clinical symptoms of patients but have limited effects on the prevention and delay of bladder fibrosis. Therefore, the management of NB patients not only requires reducing the bladder pressure to protect renal function but also requires the prevention and treatment of bladder fibrosis. However, the pathogenesis of neurogenic bladder fibrosis remains unclear.

The clinical manifestations caused by a spinal cord injury (SCI) at different segments are different. Children with spina bifida mostly have lumbosacral SCI, and the clinical manifestations are detrusor muscle non-contraction, dysuria and urinary retention. The prolonged bladder overfilling resulting from this will compress the blood vessels, decrease blood flow and eventually lead to hypoxia. Recent findings suggest that hypoxia is involved in the development of bladder dysfunction [3]. Lit et al. found that ischemia and hypoxia disturb glucose metabolism in the detrusor and cause smooth muscle degeneration and necrosis [4]. As a transcription factor, HIF-1α can promote the transcription of various genes in response to hypoxic stimulation, including those involved in EMT, pyroptosis and energy metabolism [5,6,7].

EMT plays an important role in wound healing, fibrosis and tumorigenesis and is divided into three subtypes based on its biological function. Among them, type 2 EMT is involved in tissue regeneration and fibrosis and is characterized by the differentiation of epithelial cells into new fibroblast-like cells in the mesenchyme [8]. Pyroptosis is a new type of programmed cell death (PCD) characterized by cell swelling, lysis and an inflammatory response. A number of studies have indicated that pyroptosis is involved in the occurrence and progression of fibrosis and inflammation [9,10]. As a transcription factor, HIF-1α appears to be critically involved in pyroptosis. However, whether HIF-1α induces bladder fibrosis through activation of EMT and pyroptosis is not clear.

Overall, mucosal hypoxia induced by an overstretched bladder can exacerbate tissue fibrosis in several ways. EMT and pyroptosis may be involved in the development of bladder fibrosis after SCI. This programmed inflammatory cell death and the progression of differentiation into fibroblast-like cells may be regulated by HIF-1α. In this study, we investigated the expression changes in EMT- and pyroptosis-related genes in vitro and in vivo. We found that knocking down HIF-1α expression in vitro inhibited the progression of pyroptosis and EMT, thereby alleviating bladder fibrosis.

## 2. Materials and Methods

### 2.1. Animals and NB Model

This study was approved by the Ethics Committee of Laboratory Animals of the Children’s Hospital of Chongqing Medical University, Chongqing, China. Eighteen healthy specific pathogen-free (SPF) female SD rats (weighing 160–180 g, aged 8 weeks) were purchased from the Animal Research of Chongqing Medical University. All animals were maintained under SPF conditions and fed ad libitum in a temperature-, humidity- and light-controlled room. After 1 week of acclimatization, the rats were randomly divided into three groups: the control group (n = 6), the NB 3-week group (n = 6), and the NB 6-week group (n = 6). The NB rat model was created by cutting the bilateral lumbar 6 (L6) and sacral 1 (S1) spinal nerves, as previously described [11]. Briefly, after the rats were anesthetized with 20% urethane (5 mL/kg), the L6-S1 dorsal and ventral roots were exposed and severed through a dorsal median incision. The rats in the control group had only their nerve roots exposed, and no other operations were performed. Penicillin was administered continuously for 3 days after surgery. Finally, the rats were euthanized by CO_2_ inhalation, and bladder tissue samples were collected for further experiments.

### 2.2. Cystometry

Rats were anesthetized using 20% urethane (5 mL/kg) via intraperitoneal injection, and an epidural catheter was inserted into the bladder through the urethra. The other end of the catheter was connected to a peristaltic pump and an acquisition system (RM-6240; Chengdu Instrument Factory, Chengdu, China) for infusion and pressure recording via a tee connector. Bladders were infused with saline at a rate of 10 mL/h, and the parameters of bladder leak point pressure (BLPP), bladder compliance (BC) and maximum cystometry capacity (MCC) were recorded.

### 2.3. Doppler Ultrasonography

Kidney and bladder ultrasound was performed using a Philips iU22 machine (Philips Healthcare, Eindhoven, The Netherlands) in all groups of rats under anesthesia with 20% urethane (5 mL/kg).

### 2.4. BUN/Scr Analysis

The serum levels of urea nitrogen (BUN) and serum creatinine (Scr) were measured using an autoanalyzer (Cobas C701, Roche, Basel, Switzerland).

### 2.5. Histological and Immunohistochemical Staining

Bladder tissues were fixed with 4% paraformaldehyde for 24 h, embedded in paraffin, and then cut into 4 μm sections. Sections were deparaffinized, rehydrated, and then stained with hematoxylin and eosin (H&E) and Masson’s trichrome stain, according to standard protocols. A Masson staining kit was purchased from Leagene (DC0033, Beijing, China). To analyze the fibrosis level, immunohistochemistry was performed on tissue sections. Briefly, sections were deparaffinized and rehydrated and then heated in citrate buffer for antigen retrieval. After blocking with BSA, sections were incubated with primary antibodies against alpha-smooth muscle actin (1:200; Zenbio, R23450, Chengdu, China) and collagen I alpha 1 (1:200; Zenbio, 501352, Chengdu, China) overnight at 4 °C. The cells were incubated with a secondary antibody (1:200; Zenbio, 511203, Chengdu, China) for 1 h at room temperature. Then, the sections were stained with 3,3′-diaminobenzidine and counterstained with hematoxylin.

### 2.6. RNA Sequencing and Functional Enrichment Analysis

Bladder tissues of rats in the normal and 6-week model groups were sent for RNA sequencing analyses. Each group had three replicates. Samples were sequenced on an Illumina NovaSeq 6000 platform from LC-Bio (Hangzhou, China). FDR < 0.05 and Log2FC ≥ 1 were selected as the cutoff values for identifying differentially expressed genes (DEGs) in the normal and NB groups. Hypoxia-related genes (HRGs) were downloaded from the GSEA website (http://www.gsea-msigdb.org/gsea/index.jsp, accessed on 9 October 2022). Then, we took the intersection of DEGs and HRGs to obtain the differentially expressed HRGs. Gene Ontology (GO) and Kyoto Encyclopedia of Genes and Genomes (KEGG) enrichment analyses were performed on these DEGs and differentially expressed HRGs using R software (R version 4.2.0).

### 2.7. TUNEL Analysis

A One-Step TUNEL Assay Kit (Elabscience, E-CK-A320, Wuhan, China) was used for the TUNEL assay according to standard protocols. Immunofluorescence imaging was performed using an A1R confocal microscope (Nikon, Tokyo, Japan).

### 2.8. Cell Culture and Hypoxia Treatment

A human epithelial cell line (SVHUC) was purchased from Procell Co., Ltd. (Wuhan, China) and maintained in F12K containing 10% FBS (vivacell, C04001-500) and 1% ampicillin/streptomycin. Cells were cultured under normoxic conditions (21% O_2_, 5% CO_2_, 37 °C) until 60–70% confluence and then subjected to hypoxic conditions (1% O_2_, 5% CO_2_, 37 °C) in a tri-gas incubator (Thermo Scientific, Waltham, MA, USA) for 4, 8, 12 and 24 h.

### 2.9. Western Blotting

Proteins of rat bladder and SVHUC cells were extracted with RIPA (Beyotime, P0013B, Shanghai, China) supplemented with a 1% protease inhibitor cocktail. BCA methods were used to measure the protein concentrations (Beyotime, P0012, Shanghai, China). Equal amounts of protein samples were separated via SDS-PAGE and then transferred to PVDF membranes (Millipore, Burlington, MA, USA). After blocking with skim milk powder for 2 h, the PVDF membrane was incubated with primary antibodies at 4 °C overnight. The primary antibodies were diluted in a ratio of 1:1000 and listed as follows: HIF-1α (Zenbio, 382600), alpha Smooth Muscle Actin (HUABIO, ET1607-53), Collagen I (Zenbio, 501352), E-cadherin (Abcam, ab231303, Cambridge, UK), N-cadherin (Cell Signaling Technology, 14215S), Vimentin (HUABIO, ET1610-39, Zhejiang, China), ZO-1 (Proteintech, 21773-1-AP, Wuhan, China), NLRP3 (Zenbio, 381207), GSDMD (HUABIO, HA721144), IL1-β (Abcam, ab283818), caspase1 (Proteintech, 22915-1-AP) and GAPDH (Zenbio, R24404). On the second day, the PVDF membrane was washed 3 times in TBST for 10 min and incubated with the corresponding secondary antibody for 2 h. Secondary antibodies, including goat anti-rabbit (Zenbio, 511203) and goat anti-mouse (Zenbio, 511103), were used at a dilution of 1:10,000. The protein signals were visualized with Super ECL (Everbright LTD., Suzhou, China) and quantified through Image Lab software (Bio-Rad, Hercules, CA, USA).

### 2.10. Cellular and Tissue Immunofluorescence

Bladder tissue sections were deparaffinized and hydrated. After blocking with BSA, the sections were incubated with primary antibodies (1:200) against HIF-1α (Zenbio, 382600), E-cadherin (Abcam, ab231303), N-cadherin (Cell Signaling Technology, 14215S), Vimentin (Zenbio, R22775), ZO-1 (Proteintech, 21773-1-AP), NLRP3 (Zenbio, 381207) and caspase1 (Proteintech, 22915-1-AP) overnight at 4 °C. SVHUC cells were cultured on coverslips in 24-well plates at a density of 60%. After treatment with hypoxia, the cells were fixed in 4% paraformaldehyde for 15 min and permeabilized with 0.05% Triton X-100. After blocking with BSA, the cells were incubated with primary antibodies (1:200) against HIF-1α (Zenbio, 382600), collagen I (Zenbio, 501352), E-cadherin (Abcam, ab231303), Vimentin (Zenbio, R22775), NLRP3 (Zenbio, 381207) and IL1-β (Abcam, ab283818) at 4 °C overnight. On the second day, tissue sections and cell slides were incubated with a secondary antibody (Alexa Fluor^®^ 488 or Alexa Fluor^®^ 594) at room temperature in the dark for 45 min. Then, the sections were stained with DAPI for 10 min.

### 2.11. Scanning Electron Microscopy (SEM)

The cells were fixed with 5% glutaraldehyde for 1 h and dehydrated with gradient ethanol and hexamethyldisilazane. After treatment with an ion-sputtering coating, the samples were photographed with an electron microscope.

### 2.12. Lactate Dehydrogenase (LDH) Release Assays

The level of LDH released in the cell culture supernatant and serum was measured using an LDH assay detection kit (Elabscience, E-BC-K766-M, Wuhan, China) according to the manufacturer’s recommendations.

### 2.13. Calcein-AM/Propidium Iodine (PI) Staining

Cell death was identified via a Calcein/PI Live/Dead Viability Assay Kit (Beyotime, C2015S, Shanghai, China) according to the manufacturer’s recommendations.

### 2.14. Enzyme-Linked Immunosorbent Assay for IL-1β and IL-18

The levels of IL-1β and IL-18 in serum and cell supernatants were determined using enzyme-linked immunosorbent assay kits following the manufacturer’s protocol (Elabscience, Wuhan, China).

### 2.15. siRNA Transfection

siRNA targeting human HIF-1α and the negative control were synthesized by Tsingke Biotechnology Co., Ltd (Beijing, China). The HIF-1α siRNA sequences were 5′-GCCACAUUCACGUAUAUGATT -3′ (sense) and 5′-UCAUAUACGUGAAUGUGGCTT-3′ (antisense). Transfection was performed with Lipo 3000 (Thermo Fisher, Waltham, MA, USA) and Opti-MEM (Invitrogen, Carlsbad, CA, USA).

### 2.16. Statistical Analysis

GraphPad Prism 8.0 was used for experimental data analyses. Data are presented as the mean ± standard deviation. Comparisons between groups were made using Student’s *t* test or one-way ANOVA with Bonferroni’s post-hoc test. Each experiment was repeated at least three times. * *p* < 0.05, ** *p* < 0.01 and *** *p* < 0.001 were considered statistically significant.

## 3. Results

### 3.1. The Rats Showed Bladder Dysfunction, Upper Urinary Tract Damage and Bladder Fibrosis after SCI

**Figure 1:**
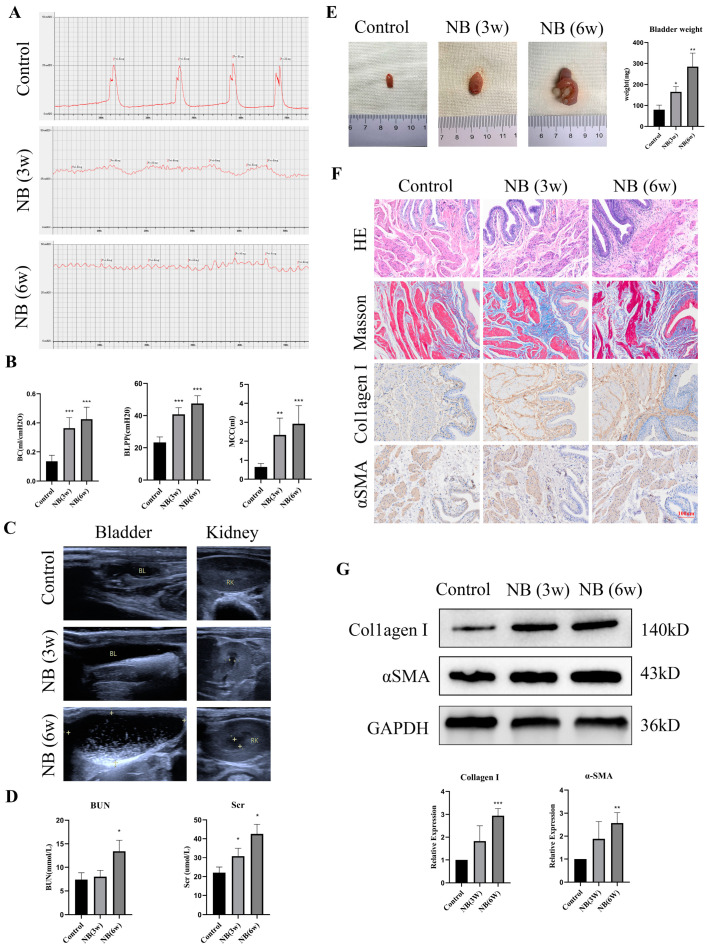
Bladder dysfunction, upper urinary tract damage, and bladder fibrosis at different times after SCI. (**A**) Cystometry in rats at different time points after SCI. (**B**) Comparison of cystometric parameters (BLPP, BC and MCC) in different groups of rats. (**C**) Ultrasound images of the bladder and kidney in different groups of rats. (**D**) Serum BUN and Scr levels in different groups of rats. (**E**) The bladder weight of rats in different groups. (**F**) HE, Masson and immunohistochemical staining of rat bladder after SCI for three and six weeks. (**G**) Collagen I and αSMA protein levels determined through Western blotting. Data are presented as the mean ± S.D. (n ≥ 3). * *p* < 0.05; ** *p* < 0.01; *** *p* < 0.001. Scale bars, 100 μm.

To assess bladder function in rats, a urodynamic examination was performed at different times after SCI. The results revealed that the rats in the control group had obvious filling and voiding phases, and the detrusor contraction peak could be seen during the voiding phase. However, the detrusor had no obvious contraction in the NB (3w) and NB (6w) groups, and the rats displayed intermittent urinary incontinence (Figure 1A). Furthermore, BLPP, BC and MCC significantly increased in rats after SCI (Figure 1B), which indicated that the rats experienced bladder dysfunction. To determine whether bladder dysfunction affects the upper urinary tract, ultrasound and biochemical examinations were performed in rats. The results showed that the rats in the NB (3w) and NB (6w) groups experienced urinary retention and hydronephrosis (Figure 1C), and the BUN and Scr results also indicated that renal function was significantly impaired (Figure 1D).

Then, the rats were sacrificed, and bladder tissues were collected and weighed. Compared with the control group, the bladder weight of the rats in the NB (3w) and NB (6w) groups increased significantly (Figure 1E). Due to long-term inflammatory stimulation, bladder stones formed in the rats of the NB (6w) group (Figure 1E). We then performed histological tests to examine pathological changes in the bladder. H&E staining showed that the NB (3w) and NB (6w) groups had obvious tissue inflammation in the epithelium area of the rat bladder sections compared to the control group (Figure 1F). Masson’s trichrome staining showed collagen accumulation with muscle bundle thickening in the NB (3w) and NB (6w) groups (Figure 1F). The results of immunohistochemistry and Western blotting found that collagen I and αSMA expression in the bladder tissue of the NB (3w) and NB (6w) groups were significantly increased, indicating an increase in the degree of fibrosis (Figure 1F,G). These results indicated that rats developed bladder fibrosis, bladder dysfunction and even upper urinary tract damage after SCI, which was consistent with the clinical manifestations of NB patients.

### 3.2. RNA Sequencing Revealed That Hypoxia Can Induce Fibrosis via EMT and the Inflammatory Response

**Figure 2:**
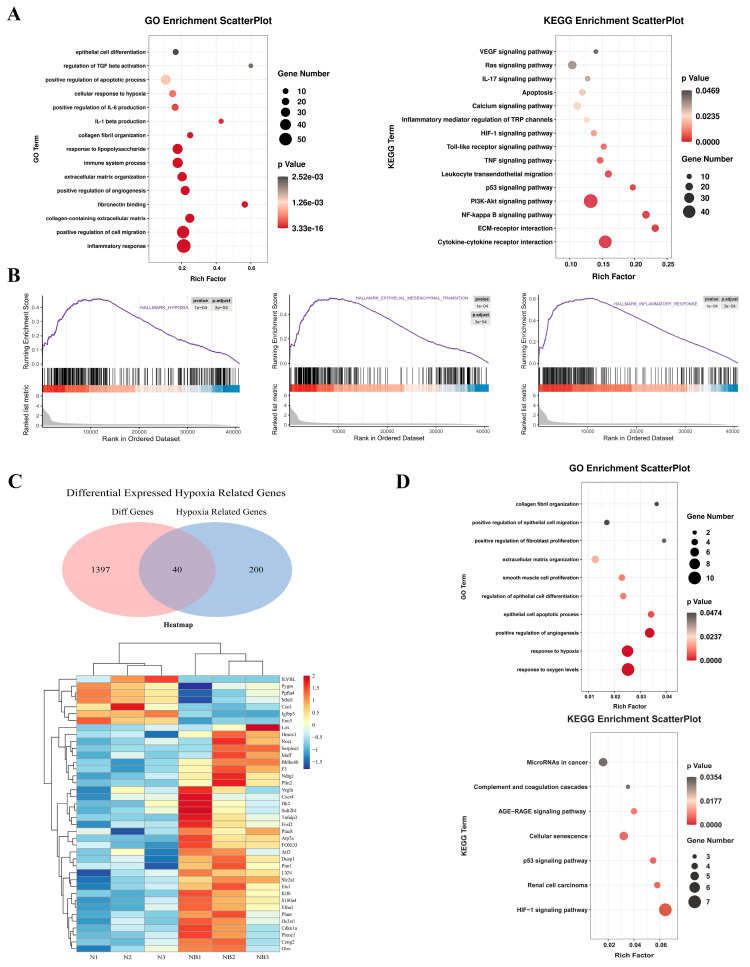
RNA sequencing assays to identify potential pathways and genes of bladder fibrosis after SCI. (**A**) GO and KEGG analyses showed enrichment of DEGs in epithelial cell differentiation, inflammatory response, HIF-1α pathway and cytokine–cytokine receptor interaction. (**B**) GSEA revealed enhanced hypoxia, EMT and inflammatory response pathway. (**C**) Heatmap of 40 differentially expressed HRGs. (**D**) GO and KEGG analyses showed enrichment of differentially expressed HRGs in response to oxygen levels and the HIF-1α pathway.

RNA-seq was used to screen DEGs between the control and NB (6w) bladder tissues to elucidate the potential mechanisms of SCI-induced bladder fibrosis. The GO results showed that DEGs were significantly enriched in epithelial cell differentiation-related and inflammatory response-related GO terms (Figure 2A). The results of KEGG analysis showed that DEGs were significantly enriched in the HIF-1α pathway and cytokine–cytokine receptor interaction (Figure 2A). Further GSEA indicated that hypoxia, EMT and the inflammatory response were upregulated (Figure 2B). HRGs were downloaded from the GSEA website. We took the intersection of DEGs and HRGs to obtain 40 differentially expressed HRGs. Next, we constructed a heatmap based on the 40 differentially expressed HRGs and found that most of them were upregulated in the NB group (Figure 2C). GO and KEGG enrichment was further conducted for the 40 differentially expressed HRGs, and the most over-enriched terms were the response to oxygen levels and the HIF-1α pathway (Figure 2D). Overall, using RNA-seq, we found that bladder fibrosis caused by SCI may involve hypoxia, EMT and the inflammatory response.

### 3.3. HIF-1α and EMT-Related Proteins Were Significantly Increased in the Bladder after SCI

**Figure 3:**
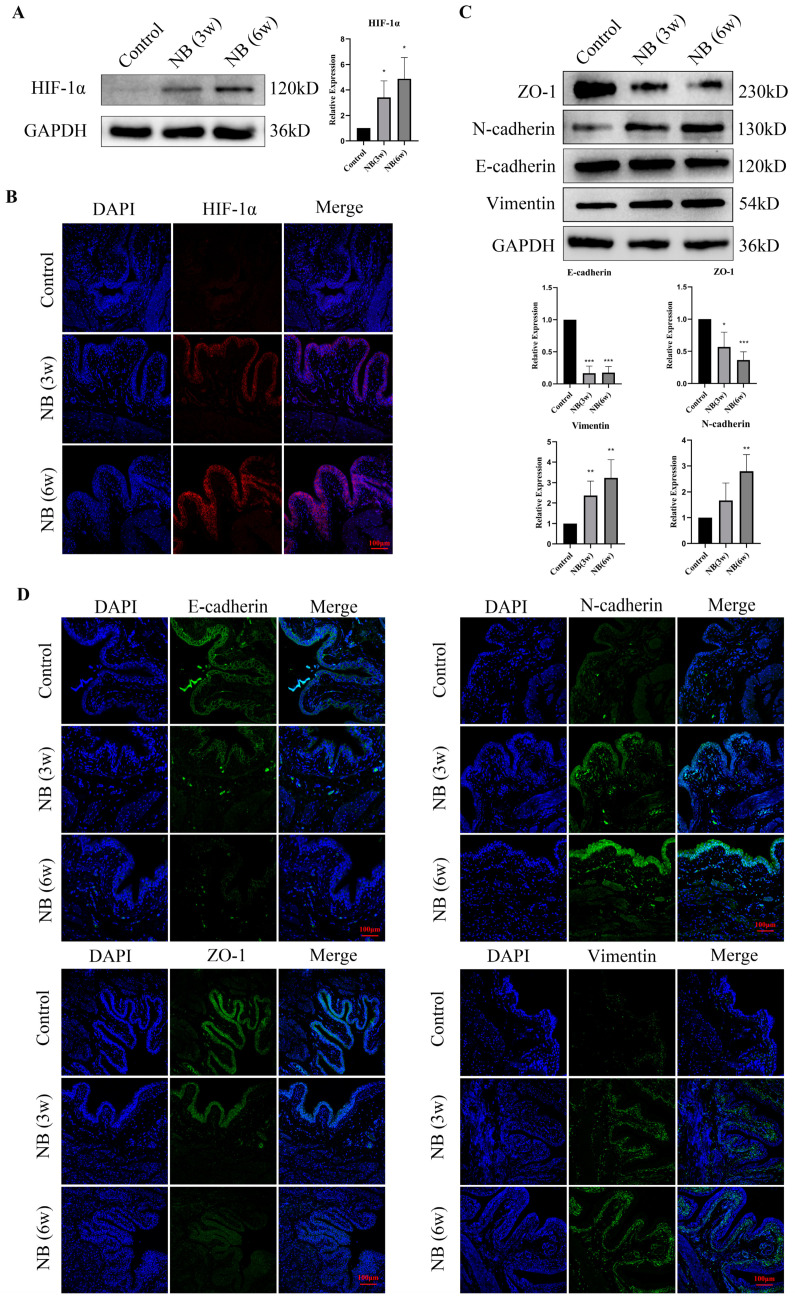
HIF-1α and EMT were involved in bladder fibrosis in the bladder after SCI. (**A**) Western blotting of HIF-1α in bladder tissue at different time points after SCI. (**B**) Immunofluorescence of bladder sections revealed that HIF-1α was upregulated in the bladder epithelial layer. (**C**) Representative Western blot bands of Vimentin, N-cadherin, E-cadherin and ZO1 and their relative optical density in bladder tissue at different time points after SCI. (**D**) Immunofluorescence of bladder sections showed upregulation of Vimentin and N-cadherin and downregulation of E-cadherin and ZO1. Data are presented as the mean ± S.D. (n ≥ 3). * *p* < 0.05; ** *p* < 0.01; *** *p* < 0.001. Scale bars, 100 μm.

According to the RNA-seq results, DEGs were enriched in the hypoxia pathway. To determine whether hypoxia occurred in bladder tissues after SCI, we examined the expression of HIF-1α protein in bladder tissue and found that the expression of HIF-1α was significantly increased in the NB (3w) and NB (6w) groups (Figure 3A). To further investigate hypoxia localization in bladder tissues, we conducted immunofluorescence on rat tissue sections. The results revealed a clear increase in HIF-1α distribution in the bladder epithelial layer compared to the control group (Figure 3B). Meanwhile, with the increase in HIF-1α, the expression of Vimentin and N-cadherin was upregulated, and the expression of E-cadherin and ZO1 was downregulated in bladder tissues after SCI (Figure 3C,D). These results indicated that hypoxia may exist in the bladder epithelial layer of rats after SCI, which induces EMT in bladder epithelial cells and eventually leads to bladder fibrosis.

### 3.4. Inflammatory Factors and Pyroptosis-Related Proteins Were Significantly Increased in the Bladder after SCI

**Figure 4:**
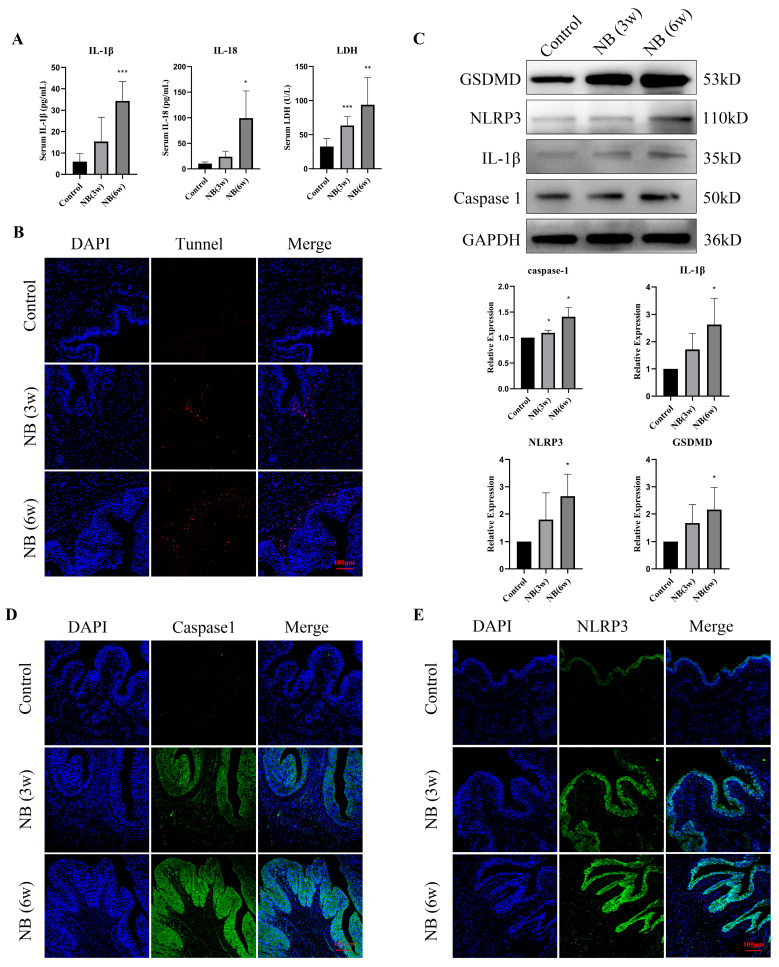
Pyroptosis was involved in the process of bladder fibrosis in the bladder after SCI. (**A**) Serum levels of IL-1β, IL-18 and LDH. (**B**) TUNEL staining of bladder tissue after SCI. (**C**) Western blot bands of NLRP3, GSDMD, IL-1β and caspase1 and their relative optical density in bladder tissue at different time points after SCI. (**D**) Immunofluorescence of bladder sections showed upregulation of caspase1. (**E**) Immunofluorescence of bladder sections showed upregulation of NLRP3. Data are presented as the mean ± S.D. (n ≥ 3). * *p* < 0.05; ** *p* < 0.01; *** *p* < 0.001. Scale bars, 100 μm.

Following SCI, the serum levels of IL-1β, IL-18, and LDH in the NB (6w) group were significantly increased compared with those in the control group (Figure 4A). Additionally, the number of TUNEL-positive cells in the NB (3w) and NB (6w) groups was significantly increased (Figure 4B). To determine whether the inflammatory response and cell damage in bladder tissue are associated with cell pyroptosis, we examined the expression of pyroptosis-related proteins. Western blot analysis showed that the expression of NLRP3, GSDMD, IL1-β and caspase1 was upregulated in the NB (3w) and NB (6w) groups (Figure 4C). Immunofluorescence results also showed significantly increased expression of caspase1 and NLRP3 in the bladder epithelial layer (Figure 4D,E). In conclusion, these results suggest an increased inflammatory response and cell damage in bladder tissue in rats after SCI, which may be associated with pyroptosis of epithelial cells.

### 3.5. Hypoxia Increased the Expression of HIF-1α and EMT-Related Proteins in Bladder Epithelial Cells

**Figure 5:**
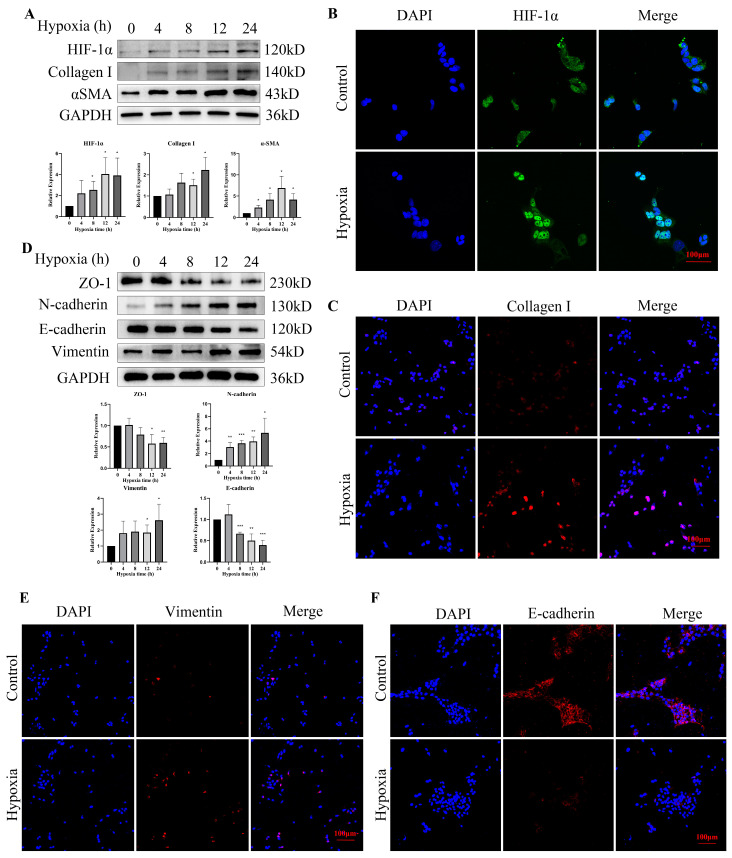
Hypoxic exposure induced the nuclear translocation of HIF-1α in epithelial cells and promoted fibrosis- and EMT-related gene expression. (**A**) Western blotting of HIF-1α, collagen I and αSMA after hypoxic exposure in epithelial cells. (**B**) Immunofluorescence of HIF-1α after hypoxic exposure for 12 h. (**C**) Immunofluorescence of epithelial cells showed upregulation of collagen I after hypoxic exposure for 12 h. (**D**) Western blotting of EMT-related proteins after hypoxic exposure in epithelial cells. (**E**) Immunofluorescence of epithelial cells showed upregulation of Vimentin after hypoxic exposure for 12 h. (**F**) Immunofluorescence of epithelial cells showed downregulation of E-cadherin after hypoxic exposure for 12 h. Data are presented as the mean ± S.D. (n ≥ 3). * *p* < 0.05; ** *p* < 0.01; *** *p* < 0.001. Scale bars, 100 μm.

As a transcription factor, HIF-1α may translocate to the nucleus to perform its biological functions once activated. We found that the expression of HIF-1α was upregulated after hypoxic exposure and translocated from the cytoplasm into the nucleus (Figure 5A,B). Additionally, with the increase in HIF-1α, the expression of collagen I and αSMA also increased (Figure 5A,C). To explore whether EMT is involved in hypoxia-induced fibrosis, we examined the expression changes in EMT-related proteins after hypoxic exposure. We found that with the hypoxia time extension, the expression of Vimentin and N-cadherin was upregulated, and E-cadherin and ZO1 were downregulated in the bladder epithelial cells (Figure 5D). Immunofluorescence staining results also demonstrated that Vimentin protein levels were increased, and E-cadherin protein levels were decreased after treatment with hypoxia for 12 h (Figure 5E,F). The expression of HIF-1α in cells treated with hypoxia for 12 h increased to a stable level compared with that of the control group. Therefore, we chose a treatment time of 12 h for the following experiments.

### 3.6. Hypoxia Stimulation Induced Pyroptosis and Promoted the Release of Inflammatory Factors in Bladder Epithelial Cells

**Figure 6:**
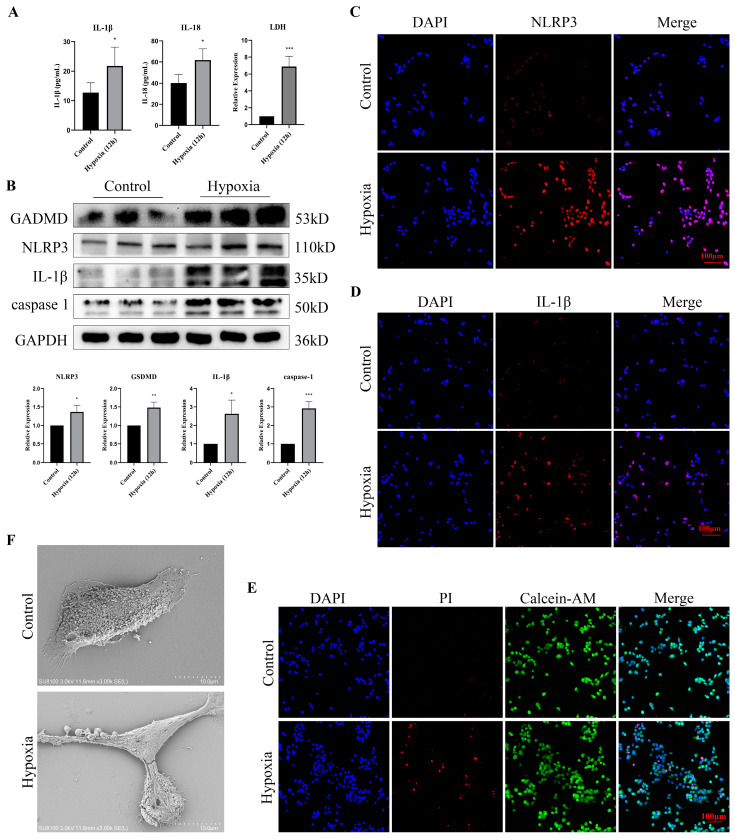
Hypoxic exposure led to epithelial cell pyroptosis. (**A**) The levels of IL-1β, IL-18 and LDH in the supernatant. (**B**) Western blot of pyroptosis-related proteins after hypoxic exposure 12h in epithelial cells. (**C**) Immunofluorescence of epithelial cells showed upregulation of NLRP3 after hypoxic exposure for 12 h. (**D**) Immunofluorescence of IL1β in epithelial cells after hypoxic exposure for 12 h. (**E**) Calcein AM/PI staining of epithelial cells after hypoxic exposure for 12 h. (**F**) Scanning electron microscopy showed that after hypoxic exposure for 12 h, the morphology of epithelial cells changed, with pyroptotic morphology characterized by pyroptotic bodies. Data are presented as the mean ± S.D. (n ≥ 3). * *p* < 0.05; ** *p* < 0.01; *** *p* < 0.001. Scale bars, 100 μm.

To clarify the relationship between hypoxia and pyroptosis, we measured the levels of inflammatory factors and the expression of pyroptosis-related proteins after hypoxia treatment. The expression of IL-1β, IL-18 and LDH in cell supernatants was significantly increased after hypoxia treatment (Figure 6A). Western blotting identified that the expression of NLRP3, GSDMD, IL1-β and caspase1 was upregulated after hypoxia treatment (Figure 6B), and the immunofluorescence of NLRP3 and IL1-β showed a similar result (Figure 6C,D). Moreover, Calcein-AM/PI staining indicated that hypoxic exposure accelerated the death of cells (Figure 6E). Using scanning electron microscopy, we observed that the epithelial cells changed to a pyroptotic morphology characterized by pyroptotic bodies after hypoxia treatment (Figure 6F). These findings indicated that hypoxic exposure induced pyroptosis in bladder epithelial cells.

### 3.7. HIF-1α Knockdown Rescued Hypoxia-Induced Pyroptosis and EMT

**Figure 7:**
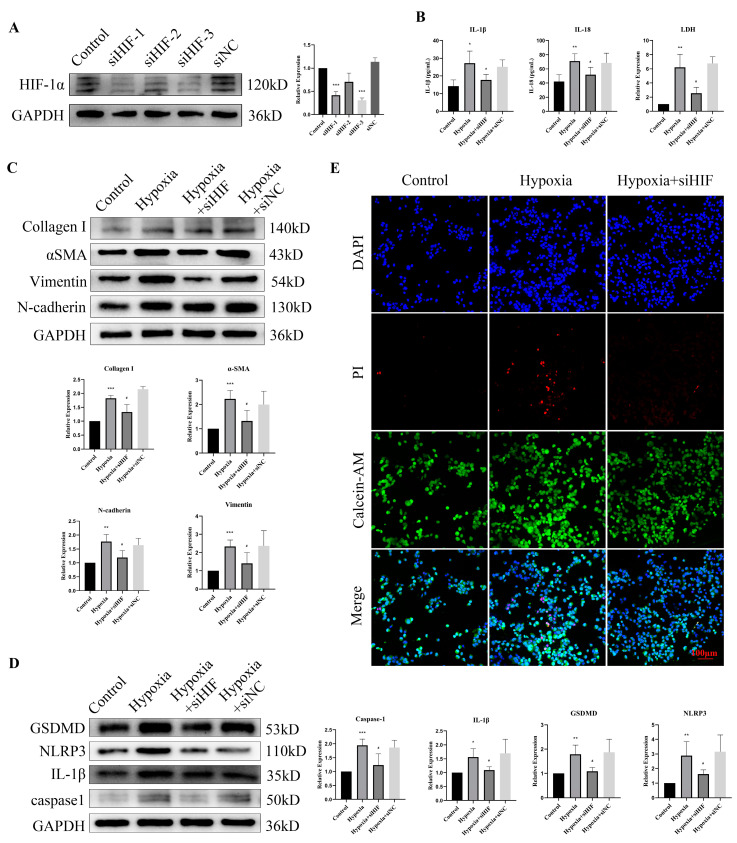
HIF-1α knockdown rescued hypoxia-induced fibrosis through EMT and pyroptosis. (**A**) The knockdown efficiency of HIF-1α in epithelial cells determined via Western blot. (**B**) HIF-1α knockdown partially reversed the increased levels of IL-1β, IL-18 and LDH in the supernatant after hypoxic exposure 12 h. (**C**) HIF-1α knockdown partially reversed the upregulation of fibrosis- and EMT-related proteins in epithelial cells after hypoxic exposure for 12 h. (**D**) HIF-1α knockdown partially reversed the upregulation of pyroptosis-related proteins in epithelial cells after hypoxic exposure for 12 h. (**E**) HIF-1α knockdown ameliorated hypoxia-induced epithelial cell death. Data are presented as the mean ± S.D. (n ≥ 3). * *p* < 0.05; ** *p* < 0.01; *** *p* < 0.001. * *p* < 0.05 compared with the control group and # *p* < 0.05 compared with the hypoxia group. Scale bars, 100 μm.

To ascertain whether hypoxia-induced fibrosis occurs via HIF-1α, we knocked down HIF-1α in bladder epithelial cells. Western blot results showed downregulation of HIF-1α expression (>70%) in cells transfected with HIF-1α siRNA (Figure 7A), and siRNA-3 was chosen for subsequent experiments. Increased IL-1β, IL-18 and LDH levels in cell supernatants under hypoxic exposure were inhibited after HIF-1α knockdown (Figure 7B). Since hypoxic exposure might induce EMT and fibrosis via HIF-1α, the expression of collagen I, αSMA, Vimentin and N-cadherin was assessed. The results showed that HIF-1α knockdown attenuated the upregulation of these protein levels due to hypoxic exposure (Figure 7C). The expression levels of NLRP3, GSDMD, IL1-β and caspase1 were significantly increased after hypoxic exposure and were reversed to normal levels by siRNA treatment (Figure 7D). Additionally, compromised cell viability, as a result of hypoxic exposure, was also improved after HIF-1α knockdown in bladder epithelial cells (Figure 7E). Taken together, these results suggest that hypoxic exposure leads to pyroptosis and EMT in a HIF-1α-dependent manner in bladder epithelial cells.

## 4. Discussion

NB is a common disease of lower urinary tract dysfunction in children and is often caused by SCI. With the progression of the disease, NB patients will exhibit varying degrees of bladder fibrosis, and the thickening and hardening of the bladder wall caused by fibrosis will further damage the upper urinary tract and jeopardize the children’s health [12]. However, the pathogenesis of SCI-induced bladder fibrosis remains unclear. Here, we first proposed that hypoxia-dependent pyroptosis and EMT are involved in bladder fibrosis in children with NB. More importantly, we found that pyroptosis in NB was mediated by the HIF-1α pathway, and HIF-1α knockdown not only alleviated hypoxia-induced cell death but also attenuated fibrosis in epithelial cells.

RNA-seq for high-throughput sequencing of cDNA has been the standard tool for gene expression analysis [13]. It can provide comprehensive transcriptome information by calculating the expression levels of different mRNAs, and discovering unknown or rare transcripts [14]. In this study, we performed a transcriptomic analysis of bladder tissues in the control and NB (6w) groups using RNA-seq data. Functional enrichment analyses showed that hypoxia, EMT and the inflammatory response were significantly enriched, indicating that these mechanisms may be involved in bladder fibrosis. Therefore, we study the relationship between hypoxia, EMT and inflammation in the following sections.

Children with NB usually have reduced bladder contractility and increased bladder compliance [15]. Due to the damage to the nerves controlling voiding, the bladder of NB patients is in a state of overfilling for long periods of time. The blood perfusion of the bladder is related to the degree of bladder filling. When the bladder is overfilled, the blood flow in the bladder decreases rapidly and causes bladder ischemia and hypoxia [16]. Ischemia and concomitant hypoxia severely impair bladder function and activate the expression of downstream genes, including HIF-1α [17]. HIF is composed of two subunits (HIF-1α and HIF-1β) which play a central role in regulating the adaptive cellular response to hypoxia [18]. In normoxic conditions, HIF-1α subunits are formed and are immediately targeted for proteolytic degradation. However, under hypoxia, HIF-1α becomes stable, easily translocates to the nucleus, and binds to HIF-1β to further regulate the expression of genes involved in glycolysis, angiogenesis, iron metabolism and other functions [19]. Previous studies found that the risk of increased HIF-1α expression in patients with overfilled bladders caused by urinary retention was four times higher than that in normal controls [20]. In this study, we found that HIF-1α was activated in rat bladder tissue after SCI, and immunofluorescence showed that the activation of HIF-1α mainly occurred in bladder epithelial cells, indicating that hypoxia may exist in the bladder epithelial layer. These results confirmed that hypoxia-induced HIF-1α upregulation may be involved in bladder fibrosis and voiding dysfunction after SCI.

CIC is the preferred treatment for NB patients, and its main purpose is to reduce intravesical pressure [21]. CIC requires the patients to perform it on time before the bladder reaches a safe capacity, which is very difficult for children. Therefore, ischemia and hypoxia caused by bladder hypertension are inevitable. This study found that HIF-1α, as a transcription factor activated by bladder hypoxia, can increase bladder fibrosis. Therefore, inhibiting the expression of HIF-1α in the bladder tissue of patients with NB may help to alleviate the progress of bladder fibrosis and improve the prognosis of children in clinical practice.

In previous research, both EMT and hypoxia were thought to be separate events that promote the development of fibrosis. Recently, the term hypoxia-induced EMT has been proposed because the signaling pathways are interconnected [22]. Among all the signaling pathways involved in hypoxia, the HIF-1α pathway is the most important in hypoxia-induced EMT [23,24]. Studies have found that the regulation of HIF-1α on EMT may act through some EMT-related transcription factors [25]. Zhu et al. found that HIF-1α can regulate EMT through the Snail/β-catenin pathway, but in their study, EMT was induced using paraquat rather than hypoxia directly [6]. In this research, bladder epithelial cells acquired the phenotype of fibroblasts after treatment with hypoxia, which was reversed by siRNA, indicating that HIF-1α may play an important role in the hypoxia-induced EMT in bladder epithelial cells.

Pyroptosis is a new type of PCD that occurs in various cells [26,27,28]. It is a rapid process involving cell membrane rupture, water influx, cellular swelling, osmotic lysis and the release of cell contents [29]. Pyroptosis can be mediated by the canonical pathway and the noncanonical pathway. In the canonical pathway, pyroptosis is regulated by inflammasome-activated caspase1 [30]. NLRP3 is the most common inflammasome; it can aggregate with ASC and recruit caspase1 to form polymers when the body is stimulated by external stimuli [31].

It was recently reported that pyroptosis has a close relationship with fibrosis. Cai et al. found that the expression levels of αSMA and NLRP3 in human liver fibrosis tissues were significantly higher than those in normal liver tissues, indicating that NLRP3 has a certain regulatory role in the process of fibrosis [32]. In addition, the expression of inflammatory factors and fibrosis-related proteins was decreased in the NLRP3^−/−^ mouse model, indicating that the knockdown of NLRP3 expression can reduce renal inflammation and fibrosis [33]. Although NLRP3 has been proven to be involved in the process of liver fibrosis, its role and mechanism in neurogenic bladder fibrosis are unclear. Our results found that the expression of pyroptosis-related proteins and inflammatory factors was significantly increased in rats after SCI. Recent studies suggest that HIF-1α plays an important role in disease development by promoting NLRP3 inflammasome activation [34]. Thus, the increased NLRP3 expression in the SCI group may be caused by the activation of the HIF-1α pathway due to bladder tissue hypoxia after overfilling. To confirm this conjecture, bladder epithelial cells were exposed to hypoxia. We found that pyroptosis-related proteins also increased with the increased expression of HIF-1α after hypoxia treatment and were reversed to normal levels in epithelial cells through the siRNA technique. The epithelial cells were changed to a pyroptotic morphology characterized by pyroptotic bodies after hypoxia treatment, shown via scanning electron microscopy. The above studies demonstrated that HIF-1α activated pyroptosis and released a variety of inflammatory factors, resulting in bladder fibrosis after SCI. Nevertheless, we did not further investigate the relationship between pyroptosis and EMT activation in this study. Wang et al. found that NLRP3 can induce EMT by promoting the TGF-β signaling pathway and R-Smad activation in renal tubular epithelial cells [35]. Thus, further studies are needed to investigate whether NLRP3 activates EMT in the same way in neurogenic bladder fibrosis.

There are some limitations to this study. First, only female rats were used in this study to improve the survival rate of the model, which may have caused a gender bias. Second, we only knocked down HIF-1α expression in bladder epithelial cells, and further studies are needed to verify our findings in vivo, such as using transgenic or knock-in mouse models. Thus, we plan to use a mixed-gender population and knock-in mouse models in follow-up studies to sufficiently elucidate the influence of HIF-1α in neurogenic bladder fibrosis.

In conclusion, we found that EMT and pyroptosis were involved in the development of SCI-induced bladder fibrosis via the HIF-1α pathway. Thus, the inhibition of the HIF-1α pathway may serve as a potential target for the development of new medicines to improve SCI-associated bladder fibrosis.

## Data Availability

The data presented in this study are available on request from the corresponding author. The data are not publicly available due to privacy.
